# Research on Partial Discharge Acoustic Emission Sensing Using Fiber Optic Sagnac Interferometer Based on Shaft–Type Multi–Order Resonant Mode Coupling

**DOI:** 10.3390/mi17020228

**Published:** 2026-02-10

**Authors:** Qichao Chen, Mengze Xu, Zhongyuan Li, Cong Chen, Weichao Zhang

**Affiliations:** 1 School of Electrical and Electronic Engineering, Harbin University of Science and Technology, Harbin 150080, China; 18804612630@163.com (M.X.); cchen@cpibj.com.cn (C.C.); weichao.zhang@hrbust.edu.cn (W.Z.); 2 Electric Power Research Institute, State Grid Heilongjiang Electric Power Co., Ltd., Harbin 150030, China; li8zhongyuan@126.com; 3 China Power Huachuang (Suzhou) Electricity Technology Research Co., Ltd., Suzhou 215123, China

**Keywords:** partial discharge, acoustic emission, Sagnac interferometer, resonant modes

## Abstract

In response to the key issues of complex internal structure, significant attenuation of partial discharge (PD) ultrasound signal propagation, and low sensor sensitivity in large oil–immersed power transformers, this paper analyzes the multi–order resonant mode vibration characteristics of the shaft–type fiber optic ultrasound sensor core structure. The displacement distribution patterns of the core structure in both transverse and longitudinal resonant modes are clarified. A strategy using oblique fiber winding rings is proposed to eliminate the problems of strain cancellation and non–accumulation of displacement in transverse and longitudinal resonant modes, which are common in traditional fiber optic ultrasound sensors with parallel fiber windings. Furthermore, design principles are provided to enhance the coverage of the free end and the high–strain regions with semi–high symmetry, as well as the vector–integrated response suitable for multi–order modes. Experimental results show that, in typical PD model detection, the oblique winding sensor exhibits a more prominent response near the high–order resonances of the core, with a detection sensitivity approximately 2.5 times higher than that of the parallel winding structure, and an overall sensitivity at least 7.4 times greater than that of traditional Piezoelectric (PZT) sensors. This demonstrates that the fiber winding method is a key design parameter determining the acoustic–solid coupling efficiency and high sensitivity performance of shaft–type fiber optic interferometric PD sensors, providing a feasible path for high–reliability fiber optic sensing solutions for online monitoring of transformer partial discharges.

## 1. Introduction

Partial discharge (PD) detection is an important technique for assessing the insulation condition of large oil–immersed power transformers. Effective detection of early insulation defects caused by PD can help avoid catastrophic insulation failures [1,2,3]. Among various PD detection methods, ultrasound–based detection technology has become a key direction in both engineering applications and theoretical research due to its advantages, including strong immunity to environmental electromagnetic interference, suitability for real–time online monitoring, and high accuracy in PD source localization [4,5]. However, the internal structure of large oil–immersed power transformers is complex, and the PD ultrasound signals suffer from significant attenuation during propagation [6]. Therefore, improving the detection sensitivity of PD acoustic emission sensors has become one of the key issues in related research. For PD ultrasound signal detection inside transformers, commonly used ultrasonic sensors include piezoelectric ceramic ultrasound sensors (electrical type) and fiber optic ultrasound sensors (optical type). Piezoelectric (PZT) ceramic sensors are widely used due to their simple structure and high commercialization, but their detection sensitivity is limited, and their structural form is relatively fixed, making further optimization to improve detection performance difficult. Additionally, PZT sensors rely on electrical signal transmission, which gives them poor electromagnetic interference resistance and makes it difficult to capture weak internal PD ultrasound signals when installed on the outer wall of the transformer oil tank [7,8,9,10]. In contrast, fiber optic ultrasound sensors have significant advantages in terms of detection sensitivity, electromagnetic interference resistance, and structural design optimization, making them a hot topic in current research. Many in–depth studies have been carried out on the design and application of various types of fiber optic ultrasound sensors [11,12,13].

For the purpose of discussing different interferometric mechanisms in PD acoustic sensing, fiber optic ultrasound sensors can be loosely grouped into multi–beam and dual–beam interferometric configurations. Multi–beam interferometric sensors mainly include fiber Bragg gratings, distributed feedback fiber lasers, Fabry–Perot interferometers, and external–cavity FabryPerot interferometers; dual–beam interferometers mainly adopt Mach–Zehnder, Michelson, and Sagnac interferometric structures. Multi–beam interferometric sensors, including Fabry–Perot– and FBG–based configurations, typically rely on phase modulation within a localized resonant or reflective structure, which can provide high sensitivity but differs fundamentally from the differential phase detection mechanism of dual–beam interferometers. However, they are more sensitive to environmental temperature changes, making it difficult to ensure the long–term stability of the system in the complex and dynamic temperature field inside the transformer [14,15,16,17].

Interferometric fiber optic partial discharge sensor systems (such as Michelson, Mach–Zehnder, Sagnac, etc.) rely on long optical paths and high phase sensitivity, achieving excellent detection of weak signals [18,19]. Wang et al. constructed a PD sensing system based on a balanced Sagnac interferometer and a chaotic fiber ring laser, achieving high signal–to–noise ratio detection of high–frequency acoustic emission (AE) signals [20]. Qian et al. directly placed a Sagnac fiber sensor inside the transformer oil channel, verifying the advantage of fiber optic sensors in terms of sensitivity compared to external PZT sensors for detecting PD in the winding [21]. Liu et al. further applied fiber optic ultrasound sensing methods to ±500 kV DC cable systems, achieving ultrasonic localization of long–distance cable PD [22]. Zhou et al. used high–sensitivity fiber optic interferometric sensors and significantly reduced the detection threshold of PD acoustic emission by optimizing the fiber coil structure [19]. These studies primarily focus on optimizing the interferometric system structure (such as depolarized light sources, phase demodulation strategies, delay fiber lengths, etc.) to improve overall sensitivity and operational stability. However, the mechanical sensitization structure used to efficiently couple the bulk wave acoustic field to the fiber still mainly adopts simple linear or ring fiber coils, with limited exploration and utilization of the acoustic–solidlight multi–field coupling mechanism [23].

To improve the efficiency of the acoustic–optical conversion while maintaining electrical insulation performance and mechanical strength, researchers have started to explore coupling fiber optics with shaft/waveguide structures, utilizing the vibration modes of the shaft to amplify the ultrasonic strain. Lima et al. were the first to propose winding fiber optics on a thin–walled cylindrical shaft and adjusted the material and geometric dimensions of the shaft to achieve a relatively flat frequency response in the range of 10–50 kHz suitable for transformer PD monitoring [24]. Ghorat et al. adhered FBGs on the outer surface of a hollow PTFE shaft to construct a shaft–FBG composite PD acoustic emission sensor, enhancing the detection sensitivity around 200 kHz by optimizing the shaft diameter and wall thickness [14]. Commercial products such as Optics11’s OptimAE–PD sensor also use a fiber wound on a specific shaft structure, with finite element design enhancing the frequency response in the 20–150 kHz PD ultrasound frequency band [25]. These studies indicate that shaft structures are an effective means of enhancing the sensitivity of fiber optic ultrasound sensors, with related work focusing on optimizing shaft materials, geometric dimensions, and local structural parameters.

However, existing literature shows that most shafts are typically treated as axisymmetric “strain amplifiers,” with optimization goals primarily focused on broadening or enhancing the amplitude–frequency response in specific frequency bands. In fact, winding fiber optics on the shaft not only helps improve the sensor’s detection sensitivity but also benefits installation and protection in engineering applications [26]. However, in existing studies, optimization of shaft–based fiber optic sensors typically focuses on shaft materials and geometric dimensions, with fiber coils often arranged in simple parallel forms on the shaft’s outer surface, neglecting the combined effects of the shaft’s vibration modes on the fiber coil’s stress and deformation response. Especially under high–frequency acoustic signals, the shaft may excite high–order resonant modes. According to solid mechanics principles, the strain amplitude of a solid at resonance reaches its maximum under constant external load. Therefore, while utilizing the shaft structure to enhance the fiber coil’s acoustic field coupling effect, it is also necessary to systematically study the coupling and winding methods between the fiber coil and the shaft to maximize the sensitivity gain brought by the high–response characteristics of the shaft’s resonant modes.

This paper addresses the key issues of complex internal structure, severe attenuation of PD ultrasound signal propagation, and low sensor detection sensitivity in large oil–immersed power transformers. By analyzing the multi–order resonant mode vibration response characteristics of shaft structures, the paper proposes replacing the traditional parallel winding of fiber coils on shaft–based fiber optic ultrasound sensors with oblique fiber winding. This change eliminates the issues of strain cancellation and displacement non–accumulation in transverse and longitudinal resonant modes that occur with parallel winding fiber coils. It enhances the strain amplification effect of the shaft structure on the fiber coil wound on its outer surface. Additionally, based on the alternating distribution of nodal and antinodal points in the transverse and longitudinal resonant modes of the shaft structure, design principles are provided for multi–order mode applications. In this study, 3D printing technology was used to fabricate fiber optic ultrasound sensors with identical shaft structure dimensions for both parallel and oblique winding fiber configurations, and frequency response and typical PD model tests were conducted on the sensors.

## 2. Interference Principle of the Sensor and Analysis of Mandrel Resonant Modes

### 2.1. Basic Principle of Sagnac Fiber–Optic Interference

**Figure 1:**
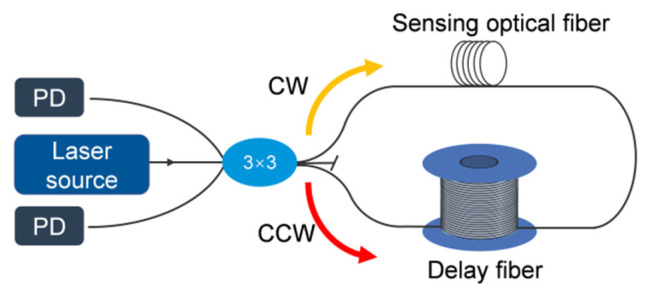
Schematic illustration of the Sagnac fiber–optic interferometric sensing system.

As shown in Figure 1, the Sagnac interferometric sensing and detection system consists of a broadband light source, a photodetector, a 3 × 3 fiber coupler, and a delay fiber. The light emitted from the source is split by the fiber coupler into two beams propagating in the clockwise (CW) and counterclockwise (CCW) directions. These two beams travel along a closed optical path and recombine to generate interference, which is subsequently converted into an electrical signal by the photodetector. In the absence of ultrasonic excitation, the CW and CCW beams experience identical optical path lengths, differing only in their propagation directions. As a result, the phases of the two beams remain identical, and the interferometric phase difference is zero. When an ultrasonic signal is applied to the sensing fiber, a time delay is introduced between the arrival of the CW and CCW beams at the sensing unit (probe) due to the presence of the delay fiber in the Sagnac loop. This time delay causes the two counter–propagating beams to be modulated by the ultrasonic signal at different moments, thereby inducing a non–reciprocal phase difference.

For the Sagnac interferometer, the two optical signals produced by the 3 × 3 coupler have intensities *I_1_* and *I_2_*, respectively, and the resulting interference intensity can be written as [27]:

(1) $$I=I_{1}+I_{2}+2\sqrt{I_{1}I_{2}}\cos\varphi$$

where *φ* denotes the phase difference between the two interfering beams, given by:

(2) φ=2πfτ

where *f* denotes the optical frequency, and *τ* represents the time delay difference between the two optical beams.

When the refractive indices of the sensing fiber and the reference fiber are identical and equal to *n*. Here, *n* denotes the effective refractive index of the guided optical mode in the fiber. The following relation holds:

(3) $$\tau =\frac{nL_{f}}{c}$$

where *L_f_* denotes the effective length of the sensing fiber section that is acoustically perturbed and contributes to the interferometric phase modulation, and *c* is the speed of light in vacuum. The interferometric phase difference *φ* can be expressed as:

(4) $$\varphi =\frac{2\pi fnL_{f}}{c}=\beta L_{f}$$

where *β* denotes the propagation constant.

External ultrasonic excitation induces variations in the interferometric phase by altering the length, refractive index, and diameter of the fiber in the sensing system. Among these factors, the effects of refractive index and diameter variations are significantly smaller than that of the fiber length change and can therefore be neglected. Following the classical theory of interferometric fiber–optic sensors [28,29], the ultrasonic–induced phase variation can be expressed as:

(5) $$\Delta \varphi =2\beta \Delta L_{1}+2\beta \Delta L_{2}=\Delta \varphi_{1}+\Delta \varphi_{2}$$

where Δ*φ_1_* represents the phase modulation induced by the radial expansion and contraction of the mandrel, and Δ*L_1_* denotes the corresponding length variation in the mandrel caused by the applied acoustic pressure. Δ*φ_2_* denotes the phase modulation resulting from the direct interaction between the optical fiber and the acoustic wave, while Δ*L_2_* represents the length variation in the fiber itself under external acoustic pressure.

From the above analysis, it can be concluded that the two counter–propagating beams in the Sagnac interferometer travel along the same optical path in opposite directions, which inherently provides high system robustness. This configuration effectively suppresses the influence of dynamic temperature variations inside the transformer on the sensing system.

Assume that the partial–discharge ultrasonic signal is $y\left(t\right)=A_{0}\sin\left(\omega_{0}t+\varphi_{0}\right)$. Since the PD ultrasonic signal is a short–duration transient signal, the i–th component (pulse) within the transient can be expressed as follows:

(6) $$y\left(t\right)=\sum_{i}^{N}A_{i}\sin\left(\omega_{i}t+\varphi_{i}\right)$$

Accordingly, the phase variations experienced by the clockwise (CW) and counterclockwise (CCW) beams caused by the PD ultrasonic signal during a single traversal of the delay fiber *L_D_* can be written as:

(7) $$y_{1}\left(t\right)=\sum_{i}^{N}A_{i}\left\{\sin\left[\omega_{i}\left(t-t_{a}\right)+\varphi_{i}\right]-\sin\left[\omega_{i}\left(t-t_{b}\right)+\varphi_{i}\right]\right\}$$

(8) $$y_{2}\left(t\right)=\sum_{i}^{N}A_{i}\left\{\sin\left[\omega_{i}\left(t-t_{c}\right)+\varphi_{i}\right]-\sin\left[\omega_{i}\left(t-t_{d}\right)+\varphi_{i}\right]\right\}$$

where $t_{a}=\left(L_{D}+L_{1}\right)\frac{n}{c}$, $t_{b}=\left(2L_{2}+L_{1}\right)\frac{n}{c}$, $t_{c}=L_{1}\frac{n}{c}$, $t_{c}=L_{1}\frac{n}{c}$, where *L* = *L*_1_ + *L*_2_. Then, the phase difference Δy(t) between the clockwise (CW) and counterclockwise (CCW) beams can be expressed as:

(9) $$\begin{array}{cc} \Delta y\left(t\right) & =y_{1}\left(t\right)-y_{2}\left(t\right)\\ & =4\sum_{i=1}^{N}\left\{A_{i}\sin\left(\omega_{i}\frac{L_{D}n}{2c}\right)\cos\left(\omega_{i}\frac{L_{2}n}{c}\right)\cos\left[\omega_{i}\left(t-\frac{\left(L_{D}+2L\right)n}{c}\right)+\varphi_{i}\right]\right\} \end{array}$$

By setting $\sin\left(\omega_{i}\frac{L_{D}n}{2c}\right)=0$ in the above expression, it follows that $\omega_{i}=\frac{2k\pi c}{L_{D}n}$. Given that $\omega_{i}=2\pi f_{i}$, the relationship between the delay fiber length *L_D_* and the PD ultrasonic signal frequency can be obtained as:

(10) $$f_{i}=\frac{kc}{nL_{D}}$$

According to the above equation, within a certain range, the frequency *f_i_* of the partial–discharge ultrasonic signal is inversely proportional to the length of the delay fiber *L_D_*. Therefore, to ensure that the fiber–optic ultrasonic sensor provides a sufficiently wide detection bandwidth over the frequency range of 20–200 kHz, where PD ultrasonic signals are widely distributed, the delay fiber length is selected as 1.5 km in this study [18,19,21].

### 2.2. Analysis of Resonant Characteristics of a Mandrel–Coupled Fiber–Optic Sensor

**Figure 2:**
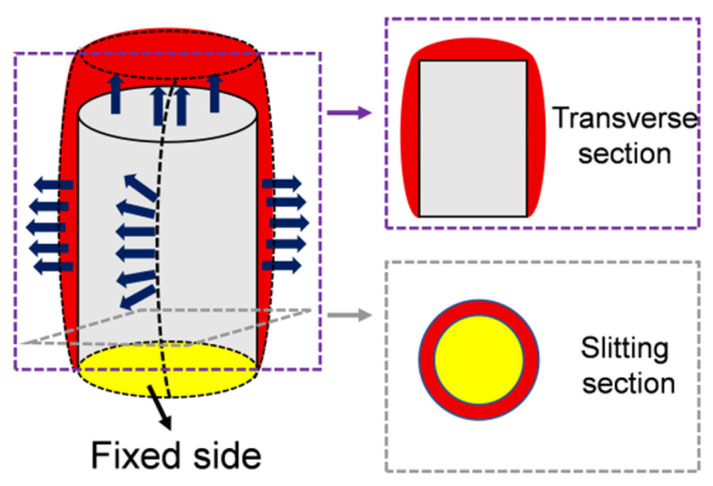
Schematic illustration of the longitudinal and transverse vibrations.

The following mechanical analysis is based on classical elasticity and vibration theory and is introduced to establish the relationship between mandrel vibration modes and fiber strain distribution. Using a mandrel as the substrate for fiber winding not only enhances the structural stability of the sensing probe but also facilitates directional design and optimization of the fiber–optic sensing structure. This allows the practical applicability of the sensor in industrial scenarios to be improved from multiple perspectives. In addition, by taking advantage of the high resonant response of the mandrel structure, the sensitivity of the fiber–optic ultrasonic sensor to weak and high–frequency partial–discharge (PD) ultrasonic signals can be significantly enhanced. The mandrel substrate is a solid cylindrical rod. When subjected to external acoustic excitation, it may exhibit multiple vibration modes, including longitudinal, transverse, and torsional vibrations. Under typical operating conditions, where the acoustic pressure generated by transformer partial discharges is relatively low, the vibration displacement of the mandrel is much smaller than its characteristic dimensions. As a result, the mandrel material can be assumed to operate within the small–strain linear elastic regime. Moreover, the contribution of torsional vibration modes to the axial strain of the optical fiber is minimal. Therefore, only longitudinal and transverse vibration modes are considered in this study. Considering the mechanical constraints under practical installation conditions, one end of the mandrel is rigidly fixed to the mounting base, while the other end is free. Accordingly, the mandrel is modeled with a classical clamped–free cantilever boundary condition, as shown in Figure 2.

For the mandrel structure, the vibration condition encountered in engineering applications corresponds to a typical single–clamped configuration. Let the length of the mandrel rod be denoted as *H*, with the axial direction defined as the *x*–axis. The axial displacement is represented by *u(x,t)*, while the transverse displacement is denoted by *w(x,t)*, where t is time. For the clamped end of the cantilever, located at *x* = 0, the boundary conditions can be expressed as [30]:

(11) $$\left\{\begin{array}{l} u\left(0,t\right)=0\\ w\left(0,t\right)=0\\ \frac{\partial w\left(0,t\right)}{\partial x}=0 \end{array}\right.$$

At the free end of the clamped–free cantilever (*x* = *H*), the boundary conditions are given by:

(12) $$\left\{\begin{array}{l} \sigma_{x}\left(H,t\right)=0\\ M\left(H,t\right)=0\\ Q\left(H,t\right)=0 \end{array}\right.$$

where *σ_x_* denotes the axial stress, *M* is the bending moment, and *Q* represents the shear force. For an Euler–Bernoulli beam, the conditions of zero bending moment and zero shear force at the free end can be equivalently expressed as:

(13) $$\left\{\begin{array}{l} \frac{\partial^{2}w\left(H,t\right)}{\partial x^{2}}=0\\ \frac{\partial^{3}w\left(H,t\right)}{\partial x^{3}}=0 \end{array}\right.$$

Since the mandrel is a three–dimensional elastic solid, its mechanical response can be decomposed into longitudinal vibration along the axial direction and transverse vibration perpendicular to the axis. Under the above assumptions, the governing wave equation for longitudinal wave propagation along the axial direction can be written as:

(14) $$\frac{\partial^{2}u\left(x,t\right)}{\partial t^{2}}=c_{H}^{2}\frac{\partial^{2}u\left(x,t\right)}{\partial x^{2}}$$

where *u(x,t)* denotes the axial displacement, and $c_{H}$ is the propagation velocity of the longitudinal wave, given by $c_{H}=\sqrt{\frac{E}{\rho }}$, where *E* is the Young’s modulus of the mandrel material and ρ is the material density. The standing–wave solution of the longitudinal vibration can be expressed as:

(15) $$u_{n}\left(x,t\right)=A_{n}\sin\left(k_{n}x\right)e^{j\omega_{n}t}$$

where $A_{n}$ is the displacement amplitude constant of the nth vibration mode, $k_{n}$ is the axial wavenumber, and $\omega_{n}$ denotes the angular frequency of the nth mode. By substituting the fixed–end boundary condition *u(0,t)* = 0, it can be shown that the above solution form satisfies this condition. At the free end (*x* = *H*), the axial stress vanishes. For a one–dimensional rod, the axial stress is given by $\sigma_{x}=E\frac{\partial u}{\partial x}$. Therefore, the free–end boundary condition can be written as $\frac{\partial u\left(H,t\right)}{\partial x}=0$. Substituting this condition into Equation (7) yields:

(16) $${\left.\frac{\partial u_{n}}{\partial x}\right|}_{x=H}=A_{n}k_{n}\cos\left(k_{n}H\right)e^{j\omega_{n}t}=0$$

When $A_{n}\neq 0$, the condition $\cos\left(k_{n}H\right)=0$ must be satisfied, which leads to $k_{n}H=\frac{\left(2n-1\right)\pi }{2},n=1,2,3,\ldots ,$ Therefore, it follows that:

(17) $$\omega_{n}=c_{H}k_{n}=\frac{\left(2n-1\right)\pi c_{L}}{2H}$$

Consequently, the nth–order longitudinal resonant frequency can be obtained as [30]:

(18) $$f_{H,n}=\frac{\omega_{n}}{2\pi }=\frac{\left(2n-1\right)c_{H}}{4H}=\frac{2n-1}{4H}\sqrt{\frac{E}{\rho }},n=1,2,3,\ldots$$

It can therefore be observed that, during longitudinal vibration, a mandrel rod with one end clamped and the other free exhibits multiple nodes (zero displacement) and antinodes (maximum displacement between two adjacent nodes) along its length. For the first resonant mode, the clamped end corresponds to a node, while the free end forms an antinode. In the second resonant mode, an additional node–antinode pair appears at the midspan of the rod. For the third and higher–order resonant modes, nodes and antinodes are distributed alternately along the rod, and the number of nodes and antinodes increases with increasing mode order.

When the mandrel rod undergoes transverse vibration, its dynamic behavior can be described using Euler–Bernoulli beam theory. The governing wave equation for the transverse displacement $w\left(x,t\right)$ is given by:

(19) $$\frac{\partial^{2}w\left(x,t\right)}{\partial t^{2}}+\frac{EI}{\rho S}\frac{\partial^{4}w\left(x,t\right)}{\partial x^{4}}=0$$

where *w(x,t)* denotes the transverse displacement, *I* is the second moment of area of the circular cross section, *S* is the cross–sectional area, and *EI* represents the bending stiffness. By applying the method of separation of variables in the form $w_{n}\left(x,t\right)=\varphi_{n}\left(x\right)e^{j\omega_{n}t}$, and substituting it into Equation (19), the characteristic equation can be obtained by combining the boundary conditions of the clamped–free cantilever beam as:

(20) $$\cos\left(\beta_{n}H\right)\cosh\left(\beta_{n}H\right)=-1$$

where $\beta_{n}$ is the generalized wavenumber associated with the bending vibration. By numerically solving Equation (20), the first several characteristic roots $\beta_{n}H$ can be approximated as $\beta_{1}H\approx 1.875$, $\beta_{2}H\approx 4.694$, $\beta_{3}H\approx 7.855$, …

Accordingly, the angular frequency of the nth–order bending vibration is given by:

(21) $$\omega_{B,n}=\beta_{n}^{2}\sqrt{\frac{EI}{\rho S}}\frac{1}{H^{2}}$$

The corresponding resonant frequency is given by:

(22) $$f_{B,n}=\frac{\omega_{B,n}}{2\pi }=\frac{\beta_{n}^{2}}{2\pi H^{2}}\sqrt{\frac{EI}{\rho S}},n=1,2,3,\ldots$$

**Figure 3:**
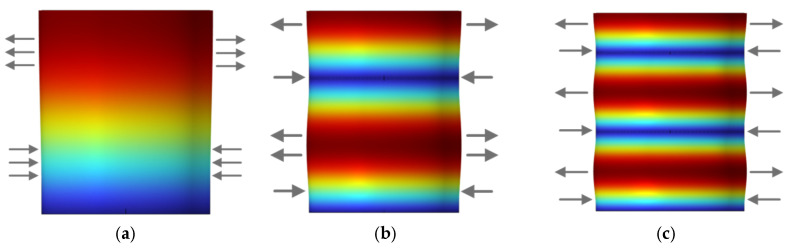

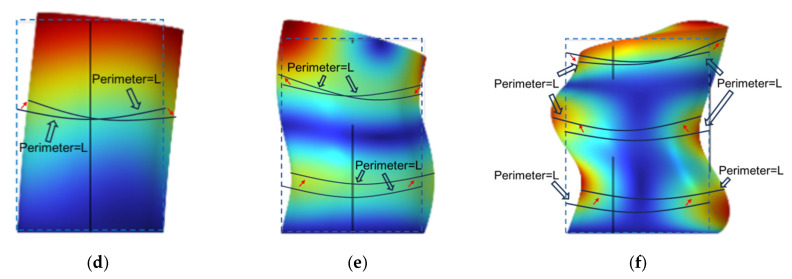
Displacement distributions of the mandrel under resonant vibration modes. (**a**–**c**) First–, second–, and third–order longitudinal vibration modes of the mandrel. (**d**–**f**) Representative bending vibration modes of the mandrel. The color contours indicate the relative displacement magnitude, and the arrows denote the displacement direction of the mandrel.

It can be observed that, compared with longitudinal vibration, the distribution of nodes and antinodes along the axial direction of a cantilevered cylindrical rod becomes more complex under bending vibration. In higher–order bending modes, the number of nodes and antinodes increases significantly, and regions of high strain tend to be more densely distributed near the free end of the rod. Based on the above analyses of longitudinal and transverse vibrations, it can be concluded that, for a mandrel rod with one end clamped and the other free, both longitudinal and bending vibration modes generate multiple nodes and antinodes along the length of the structure at different mode orders. Moreover, the regions with high vibration response are predominantly located near the free end, especially for higher–order modes. The spatial distribution of these nodes and antinodes plays a critical role in determining the effective strain perceived by optical fibers wound on the surface of the mandrel. In particular, the fiber winding configuration directly affects the extent to which high–response regions associated with different vibration modes are effectively covered. As illustrated in Figure 3, typical displacement patterns of the mandrel rod for the first several vibration modes under longitudinal and transverse excitations are presented. Figure 3a–c show the displacement distributions corresponding to the first– to third–order longitudinal vibration modes. In these cases, the mandrel experiences alternating tensile and compressive deformation along the axial direction. For conventional fiber winding schemes in which the optical fiber is wound parallel to the axial direction, a single fiber loop often spans both compressive and tensile regions simultaneously. As a result, significant strain cancellation occurs when the strain is integrated over the fiber length, leading to an insufficient effective deformation of the fiber loop. Consequently, no pronounced optical path difference is induced between the two interfering light beams, resulting in low sensing sensitivity. Figure 3d–f present the displacement distributions for the first several bending vibration modes. In this case, the dominant deformation of the mandrel is characterized by translational motion along the axial direction. For axially parallel fiber winding, the fiber loop within a given plane mainly follows this translational motion, rather than experiencing a substantial increase in circumferential deformation. Under such conditions, the displacement of the mandrel structure does not lead to an effective accumulation of fiber length variation. As a consequence, the required optical path difference between the two light beams is not sufficiently generated, giving rise to the same limitation of low sensing sensitivity as observed in the longitudinal vibration case.

**Figure 4:**
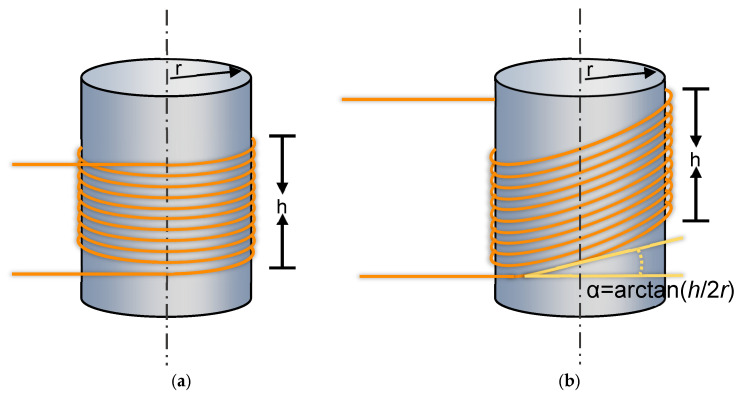
Schematic diagrams of the fiber–optic sensors: (**a**) parallel–wound fiber–optic sensor; (**b**) obliquely wound fiber–optic sensor.

Based on the above analysis, the winding configuration of the optical fiber on the mandrel surface directly determines the efficiency with which the resonant–mode–induced effective strain can be utilized by the sensor. To simultaneously exploit both the axial and radial displacement components, while mitigating strain cancellation caused by the coexistence of compression and expansion within the same plane as well as non–deformational axial translation, an oblique fiber–winding configuration is proposed in this study. In this configuration, fiber loops are wound obliquely on the outer surface of the mandrel rod, enabling asymmetric sensing of mandrel deformation. As a result, a vectorial combined response to both longitudinal and bending resonant vibration modes of the mandrel can be achieved. Moreover, under asymmetric modal deformation, a non–uniform coverage region is formed, which enhances the sensing capability for localized regions with large strain. A schematic illustration of the oblique winding structure is shown in Figure 4b. The winding angle of the optical fiber on the mandrel surface is denoted as α, which is defined as the angle between the plane of the fiber loop and the end face of the mandrel. Accordingly, α can be expressed as arctan(*h/2r*).

Based on the simulation results shown in Figure 3, it can be observed that, for both longitudinally and transversely dominated vibration modes of the mandrel structure, the displacement at the clamped boundary remains zero, while the regions with high displacement response are located near the free end of the mandrel, mainly concentrated in the upper half of the structure. Along the axial direction of the mandrel, the nodes and antinodes of both longitudinal and transverse vibration modes are approximately arranged in an alternately spaced manner. Moreover, this distribution exhibits an approximately symmetric pattern with respect to the mid–height of the mandrel, while the maximum displacement deformation still occurs at the free end. These characteristics are consistent with the high–order resonant vibration modes of a conventional cantilever beam [31]. Therefore, in order to minimize strain cancellation under coupled longitudinal–transverse vibration modes and to efficiently utilize the strain induced by single–mode vibrations of the mandrel, the oblique winding angle of the fiber loops is selected as α = arctan(*H/4r*), corresponding to an oblique winding height of *h* = *H/2*. With this configuration, the optical fiber spans both the strong–response region near the free end and the symmetric high–response region around the mid–height of the mandrel along the axial direction. As a result, a relatively large integrated equivalent strain can be obtained under resonant excitation at different mode orders. It should be noted that the objective of this study is to propose a generally applicable oblique winding design concept. Accordingly, the selection of the oblique winding height emphasizes comprehensive applicability across multiple vibration modes, rather than strict optimization for a specific mode order. This design strategy avoids excessive tuning to a particular resonance frequency and ensures stable sensitivity performance over a relatively wide frequency bandwidth, thereby providing a foundation for systematic parameter optimization in future studies.

## 3. Experimental Investigation

Since this study aims to investigate the relationship between the fiber winding configuration on the mandrel structure and the resulting sensing sensitivity, and to ensure consistency and accuracy in the fabrication of the sensor mandrels, a photosensitive resin material was selected for manufacturing the mandrel structures using 3D printing technology. The material has a Young’s modulus of 2.8 GPa, a Poisson’s ratio of 0.27, and a density of 1.1 g/cm^3^. The fabricated mandrel has a radius of 15 mm and a height of 40 mm. Based on the formulations presented in the previous section, the first four longitudinal resonant frequencies of the mandrel are calculated to be 10.1 kHz, 31.5 kHz, 48.6 kHz, and 67.4 kHz, respectively, while the first three bending resonant frequencies are 4.2 kHz, 28.3 kHz, and 71.1 kHz.

**Figure 5:**
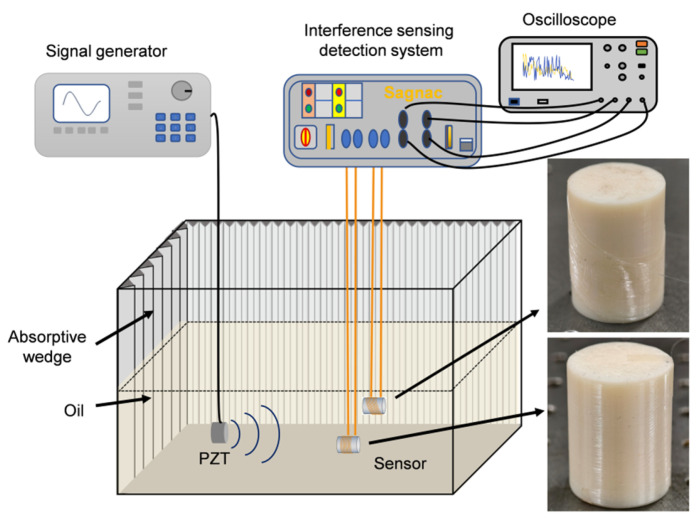
Experimental platform for sensor performance comparison.

**Figure 6:**
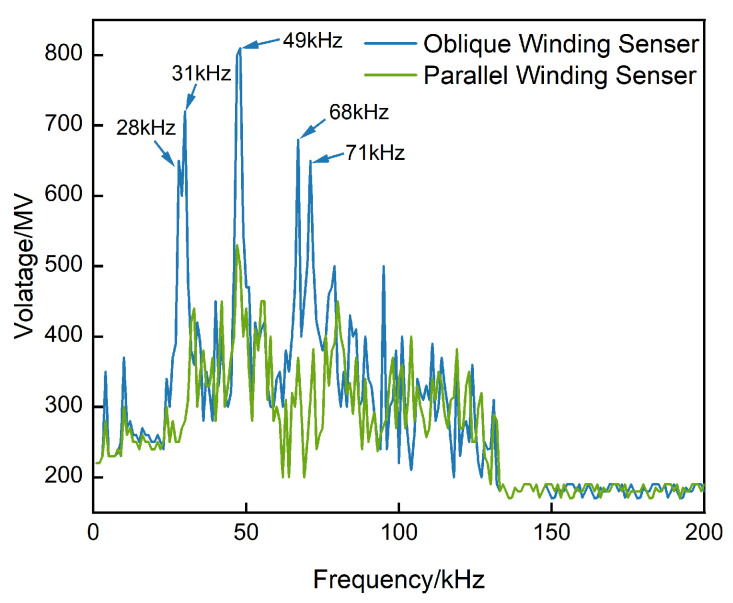
Amplitude–frequency response comparison of sensors with different winding configurations.

Considering that the intended application scenario of the proposed fiber–optic ultrasonic sensor is the interior of oil–immersed power transformers, an oil tank equipped with acoustic absorbing and reflecting materials was employed to evaluate the amplitude–frequency characteristics of fiber–optic ultrasonic sensors with two different winding configurations. The experimental setup is shown in Figure 5. A calibrated acoustic emission PZT sensor (model REF–VL) was used as the acoustic excitation source and driven by a signal generator. The amplitude–frequency response was measured over a frequency range of 0–200 kHz with a step size of 1 kHz. The measured amplitude–frequency characteristics of the sensors are presented in Figure 6.

Based on the experimental results, it can be observed that fiber–optic sensors with both winding configurations exhibit relatively low responses to the first–order longitudinal and bending resonant modes, while showing stronger responses at higher–order resonant frequencies. A certain discrepancy is found between the experimentally measured resonant frequencies and those calculated theoretically. This deviation arises because the analytical formulations presented earlier do not account for the optical fiber wound on the outer surface of the mandrel. The introduction of the fiber alters the overall mass distribution of the mandrel structure, thereby leading to shifts in the resonant frequencies.

Under identical experimental conditions, the amplitude–frequency response of the obliquely wound fiber–optic ultrasonic sensor is clearly superior to that of the parallel–wound configuration. In particular, for both longitudinal and bending resonant modes of the mandrel, the obliquely wound sensor exhibits a significant improvement in detection sensitivity. These results demonstrate that, for fiber–optic ultrasonic sensors based on mandrel structures, the fiber winding configuration plays a crucial role in determining sensing performance.

**Figure 7:**
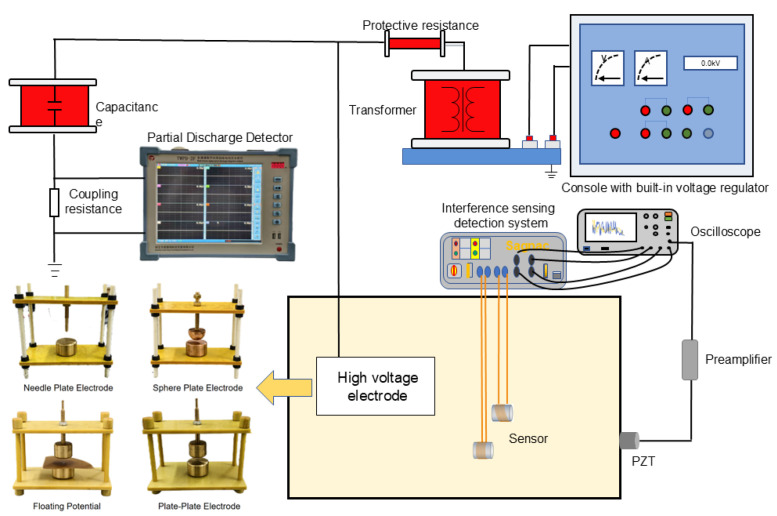
Schematic diagram of the transformer partial–discharge detection experimental platform.

To further validate the above theoretical analysis under practical partial–discharge detection conditions, a PD detection platform based on fiber–optic ultrasonic sensors was established using four typical electrode models, as shown in Figure 7.

**Figure 8:**
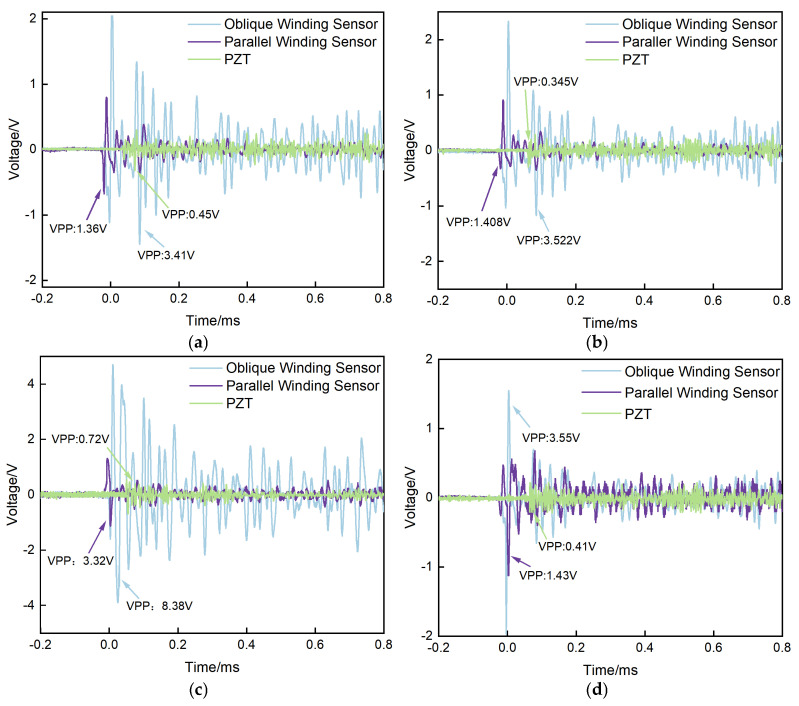
Partial–discharge detection results under different electrode configurations: (**a**) needle–plate electrode; (**b**) plate–plate electrode; (**c**) sphere–plate electrode; (**d**) floating–potential electrode.

In this study, plate–plate, needle–plate, sphere–plate, and floating–potential electrode configurations were employed as partial–discharge (PD) simulation sources to represent various PD types occurring inside power transformers. Fiber–optic ultrasonic sensors with two different winding configurations were placed in parallel inside the oil tank at equal distances from the PD source. In addition, a conventional commercial PZT sensor with a resonant frequency of 150 kHz was used as a reference sensor and as the triggering source. The experimental results are presented in Figure 8.

The experimental results indicate that, under all four electrode configurations, the output signal amplitude of the obliquely wound fiber–optic ultrasonic sensor is consistently higher than that of the parallel–wound configuration. The peak–to–peak values of the output signals are approximately 2.5 times larger, demonstrating that the oblique winding configuration provides a substantially higher detection sensitivity compared with the parallel winding approach. Furthermore, under the needle–plate, plate–plate, sphere–plate, and floating–potential electrode configurations, the peak–to–peak output signals of the obliquely wound fiber–optic ultrasonic sensor are 7.4, 10.2, 11.5, and 8.9 times higher than those of the reference PZT sensor, respectively. These results confirm that the proposed fiber–optic ultrasonic sensor exhibits a markedly higher detection sensitivity than conventional PZT sensors. However, due to the stochastic nature of the acoustic energy spectrum associated with partial–discharge ultrasonic signals, as well as the differences in amplitude–frequency characteristics between commercial PZT sensors and the developed fiber–optic ultrasonic sensor, the ratio of peak–to–peak output signals between the two sensor types shows a certain degree of variability. Based on the above experimental observations, it can be concluded that the detection sensitivity of the obliquely wound fiber–optic ultrasonic sensor is at least 7.4 times higher than that of the conventional PZT sensor.

## 4. Conclusions

Aiming at the engineering challenges associated with ultrasonic partial–discharge signal attenuation and the complex electromagnetic environment in large oil–immersed power transformers, this study developed a PD acoustic emission sensing scheme based on a Sagnac fiber–optic interferometer. Both theoretical analysis and experimental validation were conducted with a particular focus on the relationship between fiber winding configuration and the strain utilization efficiency of multi–order resonant vibration modes of the mandrel. The main conclusions can be summarized as follows:

1. An oblique winding strategy for enhanced sensitivity based on multi–order resonant mode coupling of the mandrel was proposed and validated. To address the strain cancellation caused by conventional parallel winding across tensile and compressive regions during longitudinal vibration, as well as the difficulty in effectively converting translational components into circumferential strain accumulation during bending vibration, an obliquely wound fiber–loop configuration on the mandrel surface was proposed. By enabling asymmetric coverage and a vectorial combined response to deformation, the proposed strategy enhances the sensing efficiency of both longitudinal and transverse multi–order resonant modes from a mechanistic perspective.
2. Experimental results demonstrate that oblique winding significantly outperforms parallel winding in both amplitude–frequency response and PD detection sensitivity. A mandrel–based fiber–optic ultrasonic sensor fabricated using 3D printing technology was evaluated through amplitude–frequency response measurements and PD detection experiments employing four typical PD models. The results show that, under identical conditions, the obliquely wound sensor exhibits a more pronounced response near higher–order resonant modes of the mandrel, achieving a detection sensitivity approximately 2.5 times higher than that of the parallel–wound configuration.
3. A substantial sensitivity advantage over conventional PZT sensors was achieved under representative PD conditions. For plate–plate, needle–plate, sphere–plate, and floating–potential electrode PD sources, the peak–to–peak output signals of the obliquely wound fiber–optic sensor reach 7.4, 10.2, 11.5, and 8.9 times those of a conventional PZT sensor, respectively. Based on the overall experimental results, the detection sensitivity of the proposed sensor can be considered to be at least 7.4 times higher than that of the traditional PZT sensor.

In summary, for mandrel–based fiber–optic interferometric PD acoustic emission sensors, the fiber winding configuration is not a secondary fabrication parameter, but rather a critical design factor that directly determines the strain accumulability of multi–order resonant modes and the efficiency of acousto–optic conversion. Future work will focus on the joint optimization of mandrel structural parameters and sensor sensitivity and bandwidth, with the aim of achieving more controllable broadband high–sensitivity performance, as well as advancing long–term stability assessment and engineering packaging validation under real transformer operating conditions.

## Data Availability

The original contributions presented in this study are included in the article. Further inquiries can be directed to the corresponding author.

## References

[1] Ilkhechi H.D., Samimi M.H. (2021). Applications of the Acoustic Method in Partial Discharge Measurement: A Review. IEEE Trans. Dielectr. Electr. Insul..

[2] (2019). IEEE Guide for the Detection, Location and Interpretation of Sources of Acoustic Emissions from Electrical Discharges in Power Transformers and Power Reactors.

[3] Sikorski W. (2019). Development of Acoustic Emission Sensor Optimized for Partial Discharge Detection in Power Transformer. Sensors.

[4] Deng J., Xiao H., Huo W., Luo M., May R., Wang A., Liu Y. (2001). Optical Fiber Sensor–Based Detection of Partial Discharges in Power Transformers. Opt. Laser Technol..

[5] MacAlpine M., Zhiqiang Z., Demokan M.S. (2002). Development of a Fibre–Optic Sensor for Partial Discharges in Oil–Filled Power Transformers. Electr. Power Syst. Res..

[6] Kweon D.J., Chin S.B., Kwak H.R., Kim J.–C., Song K.–B. (2005). Analysis of Ultrasonic Signals by Partial Discharge and Noise from the Transformer. IEEE Trans. Power Deliv..

[7] Hang T., Glaum J., Genenko Y.A., Phung T., Hoffman M. (2016). Investigation of Partial Discharge in Piezoelectric Ceramics. Acta Mater..

[8] Meitei S.N. (2024). Partial discharge detection using piezoelectric sensors on power transformer: A review. IEEE Sens. J..

[9] Wei P., Han X., Xia D., Liu T., Lang H. (2018). Novel Fiber–Optic Ring Acoustic Emission Sensor. Sensors.

[10] Zhou Y., Liu Y., Wang N., Han X., Li J. (2022). Partial Discharge Ultrasonic Signal Pattern Recognition in Transformer Using BSO–SVM Based on Microfiber Coupler Sensor. Measurement.

[11] Ma G.M., Zhou H., Zhang M., Li C.–R., Yin Y., Wu Y.–Y. (2019). A High Sensitivity Optical Fiber Sensor for GIS Partial Discharge Detection. IEEE Sens. J..

[12] Shao M., Cao Z., Gao H., Yu D., Qiao X. (2023). Optical Fiber Ultrasonic Sensor Based on Partial Filling PDMS in Hollow–Core Fiber. Opt. Laser Technol..

[13] Liu F., Du J., Shi Y., Zhang S., Wang W. (2024). Localization of Dual Partial Discharge in Transformer Windings Using Fabry–Pérot Optical Fiber Sensor Array. Energies.

[14] Ghorat M., Gharehpetian G.B., Latifi H., Hejazi M.A., Layeghi A. (2018). Partial Discharge Acoustic Emission Detector Using Mandrel–Connected Fiber Bragg Grating Sensor. Opt. Eng..

[15] Li H., Lv B., Tian M., Huang W., Zhang W. (2024). Temperature Compensation of Fiber Optic Unbalanced Interferometers for High–Resolution Static Strain Sensing. Opt. Lasers Eng..

[16] Yu B., Kim D.W., Deng J., Xiao H., Wang A. (2003). Fiber Fabry–Perot Sensors for Detection of Partial Discharges in Power Transformers. Appl. Opt..

[17] Dong B., Han M., Sun L., Wang J., Wang Y., Wang A. (2008). Sulfur Hexafluoride–Filled Extrinsic Fabry–Pérot Interferometric Fiber–Optic Sensors for Partial Discharge Detection in Transformers. IEEE Photonics Technol. Lett..

[18] Posada–Román J., García–Souto J.A., Rubio–Serrano J. (2012). Fiber Optic Sensor for Acoustic Detection of Partial Discharges in Oil–Paper Insulated Electrical Systems. Sensors.

[19] Zhou H., Ma G., Zhang M., Zhang H.–C., Li C.–R. (2021). A High Sensitivity Optical Fiber Interferometer Sensor for Acoustic Emission Detection of Partial Discharge in Power Transformer. IEEE Sens. J..

[20] Wang L., Fang N., Wu C., Qin H., Huang Z. (2014). A Fiber Optic PD Sensor Using a Balanced Sagnac Interferometer and an EDFA–Based DOP Tunable Fiber Ring Laser. Sensors.

[21] Qian S., Chen H., Xu Y., Su L. (2018). High Sensitivity Detection of Partial Discharge Acoustic Emission within Power Transformer by Sagnac Fiber Optic Sensor. IEEE Trans. Dielectr. Electr. Insul..

[22] Liu Z., Wang Y., Chen X., Meng X., Liu X., Yao J. (2021). An Optical Fiber Sensing Method for Partial Discharge in the HVDC Cable System. Int. J. Electr. Power Energy Syst..

[23] N’cho J.S., Fofana I. (2020). Review of Fiber Optic Diagnostic Techniques for Power Transformers. Energies.

[24] Lima S.E.U., Frazão O., Farias R.G., Araujo F.M., Ferreira L.A., Santos J.L., Miranda V. (2010). Mandrel–Based Fiber–Optic Sensors for Acoustic Detection of Partial Discharges—A Proof of Concept. IEEE Trans. Power Deliv..

[25] Zadeh A.R., Piva N., Castro Heredia L. (2019). Partial Discharge Detection and Characterization with Optical Acoustic Emission Sensors. Optics11, Application Note. https://optics11.com/wp-content/uploads/2021/08/OptimAE-PD_Optics11.pdf.

[26] Bruno F.A., Janneh M., Gunda A., Kyselica R., Stajanca P., Werzinger S., Gruca G., Rijnveld N., Persiano G., Cutolo A. (2022). Fiber Optic Hydrophones for towed array applications. Opt. Lasers Eng..

[27] Koo K.P., Tveten A.B., Dandridge A. (1983). Passively Stabilized Fiber Interferometers Using (3×3) Fiber Directional Couplers. Fiber Optic and Laser Sensors I.

[28] Hocker G.B. (1979). Fiber–optic sensing of pressure and temperature. Appl. Opt..

[29] Layton M.R., Bucaro J.A. (1979). Optical fiber acoustic sensor utilizing mode–mode interference. Appl. Opt..

[30] Timoshenko S.P., Young D.H., Weaver W. (1990). Vibration Problems in Engineering.

[31] Harris C.M., Piersol A.G. (2002). Harris’ Shock and Vibration Handbook.

